# Editorial: Rising stars in systems microbiology: 2021

**DOI:** 10.3389/fmicb.2023.1137550

**Published:** 2023-02-08

**Authors:** Anil Kumar Puniya, Gareth Wyn Griffith, Emilio M. Ungerfeld

**Affiliations:** ^1^Dairy Microbiology Division, Indian Council of Agricultural Research (ICAR)-National Dairy Research Institute, Karnal, India; ^2^Department of Life Sciences, Aberystwyth University, Aberystwyth, United Kingdom; ^3^Centro Regional de Investigación Carillanca, Instituto de Investigaciones Agropecuarias, Vilcún, Chile

**Keywords:** *Burkholderia pseudomallei*, rumen bacteriome, gene regulatory network, polycystic ovary syndrome, rising stars

Identifying tomorrow's “Systems Microbiology” front-runners is critical for promoting and stimulating future research and innovation in biology. This collection highlights the high-quality research work of globally known scientists in their early academic careers. We have aimed to highlight the research conducted by the future leaders in the area of “Systems Microbiology”, as well as their theoretical, experimental, and methodological advances for solving exciting research problems.

Li M. et al. studied the association between the composition of the rumen bacteriome, the concentration of rumen metabolites, and nitrogen utilization efficiency in dairy cows. Cows utilizing nitrogen more efficiently had higher propionate molar percentage, lower rumen bacterial diversity, and higher relative abundances of three bacterial taxa (*Succinivibrionaceae*_UCG_001, uncultured_*Selenomonadaceae*, and *Acidaminococcus* spp.). This study generates significant insights toward the improvement of nitrogen utilization efficiency in dairy cows, which has important consequences for production, cost-effectiveness, and the environment.

The paper on the genomic diversity of the clinical strains of *Burkholderia pseudomallei* and its resistance and virulence factors by Shafiq et al. showed *B. pseudomallei* to be a highly-infectious microbe in humans and animals causing melioidosis. The researchers studied the phylogenetic and evolutionary relationships among different strains of *B. pseudomallei* using genomic diversity, resistance determinants, and molecular epidemiology. The researchers discovered new virulence factors and mutations among different strains of *B. pseudomallei* causing pneumonia, neurological diseases, abscesses and lesions in the skin. The researchers also reported two novel sequences (i.e., ST1774 and ST1775) that do not match other strains of *B. pseudomallei*.

The study by Romero et al. on the reconstruction of archaeal and bacterial genomic regulatory networks on the basis of homology is important to understand the effects of various environmental stimuli on gene expression, as a key metabolic activity for microbes to adapt to environmental changes. The authors inferred the gene regulatory networks of archaeal and bacterial genomes by comparing them with the six organisms with known regulatory interactions. The gene regulatory network of these six model organisms was expanded using transcription-unit assignments and orthologs inferences. They demonstrated the most significant findings from these inferences through several examples and also by calculating the topological metrics of such networks. As a result, this approach is a valuable resource for reconstructing the regulatory network of interactions occurring within archaeal and bacterial domains which can be further combined with the globally available expression data for these organisms to improve global interaction data models. Inferences of gene regulatory networks are critical to understanding bacterial metabolism. Finally, the authors provided readers with a website to analyze and download all the network data.

The paper by Li G. et al. examined the differences in composition of gut microbiota fecal fatty acids in patients suffering from polycystic ovary syndrome (PCOS) compared to healthy subjects. Specific metabolites present in the serum and fatty acids produced by the intestinal fecal microflora may modulate the onset and progression of PCOS. The specific intestinal microbiota in PCOS patients has been reported to vary by body mass. By using clinical-index detection techniques of stool and serum samples from PCOS patients, it was possible to elucidate potential correlations between altered gut microbiota and inflammatory markers, as well as short-chain fatty acid metabolism and gut permeability markers. The gut microbiota of patients with different body types and PCOS has never been thoroughly characterized before this study. These discoveries will provide a strong framework for understanding the etiology of PCOS and potentially guide future medical interventions in its treatment, for example fecal bacteria transplantation.

Research innovations resulting from research presented in “*Rising stars in systems microbiology: 2021*” are yet to reach fruition; however, the articles published in this Research Topic point toward exciting and promising future directions. We hope that the topics will inspire future generations of scientists to continue advancing these areas of research.

## Author contributions

AP, GG, and EU contributed to the writing and editing of the manuscript. All authors contributed to the article and approved the submitted version.

